# COL5A2 Inhibits the TGF-β and Wnt/β-Catenin Signaling Pathways to Inhibit the Invasion and Metastasis of Osteosarcoma

**DOI:** 10.3389/fonc.2022.813809

**Published:** 2022-02-24

**Authors:** Yan-Long Han, Dan Luo, Kakeng Habaxi, Julaiti Tayierjiang, Wei Zhao, Wei Wang, Wumaierjiang Aikebaier, Li Wang

**Affiliations:** ^1^ Department of Bone and Joint Surgery Center, People’s Hospital of Xinjiang Uygur Autonomous Region, Urumqi, China; ^2^ Department of Respiratory and Critical Care Medicine, People’s Hospital of Xinjiang Uygur Autonomous Region, Urumqi, China

**Keywords:** osteosarcoma, COL5A2, TGF-β, Wnt/β-catenin, tumor metastasis

## Abstract

Osteosarcoma is the most common skeletal malignancy and is the second leading cause of cancer death in adolescents. Its highly aggressive nature and high propensity to metastasize lead to an extremely poor prognosis for patients with osteosarcoma. Therefore, finding a suitable treatment has become a matter of urgency. In this study, we first divided the samples into metastatic and non-metastatic groups using the Target database and obtained 1136 differentially expressed genes (DEGs) using differential analysis. A PPI network was constructed to analyze the network of action relationships among DEGs, and the top 10 genes were derived using the MCC algorithm in Cytoscape software. A risk scoring system for 10 key genes was constructed using the LASSO-COX prognostic risk model, and genes associated with osteosarcoma prognosis were screened based on multifactorial COX. COL5A2 gene was highly expressed in metastatic osteosarcoma and led to a poor prognosis. Furthermore, qRT-PCR and immunofluorescence assays confirmed the high expression of COL5A2 in human osteosarcoma cells. CCK-8 assay and scratch WB was used to determine whether the downregulation of COL5A2 expression inhibits the TGF-β signaling and Wnt/β-Catenin signaling pathways. In this study, we screened COL5A2 for prognostic relevance to osteosarcoma through bioinformatics analysis and demonstrated that COL5A2 inhibited osteosarcoma invasion and metastasis by suppressing the TGF-β signaling and Wnt/β-Catenin signaling pathways.

## Introduction

Osteosarcoma (OS) is the most common primary skeletal malignancy (1). Both adolescents and older adults have a peak incidence, which results in a bimodal distribution of osteosarcoma incidence (2). Various factors contribute to the increased incidence of osteosarcomas, such as race and gender, but younger age is the most common risk factor (3). Osteosarcoma has been reported to account for over 50% of malignant bone tumors in children and is the second leading cause of cancer death in adolescents (4). The commonly used treatment in the past was surgical resection, but this approach did not improve the survival of patients with metastatic osteosarcoma (5). Modern combination treatments such as intensive chemotherapy have now led to a 35%–50% increase in five-year survival in patients with localized disease (6, 7). However, the five-year survival rate remains low for the patient population with metastatic lesions (8). The main reasons for the low long-term survival of patients with osteosarcoma are the highly aggressive and early systemic metastatic nature of the osteosarcoma (9). Approximately 20% of patients are identified as metastatic at diagnosis, and less than 15% of patients without metastatic disease are treated for five-year survival (10). In contrast, pulmonary metastases are the most prominent type of distant metastasis in osteosarcoma, with a five-year survival rate of no more than 30% (2).

Distant metastasis is a predictor of poor tumor prognosis, and specific metabolic pathways can promote secondary tumor formation through the induction of signaling pathways (11). During the treatment of cancer patients, tumor progression was found to be accelerated when treatment was stopped and the epithelial-mesenchymal transition (EMT) process was induced (12). EMT is a plastic cellular process that is associated with cancer cell invasion and metastasis (13). Moreover, EMT is a hallmark of metastatic cancer; there are multiple important signaling pathways involved in accelerating the EMT process and tumor metastasis, including the transforming growth factor-β1 (TGF-β) signaling and Wnt/β-Catenin signaling pathways (14). TGF-β is a secreted cytokine that plays an important role in EMT, cell biological progression, etc. (15). TGF-β acts as a tumor suppressor in the early stage of tumorigenesis, while the subsequent TGF-β signaling pathway can effectively induce the EMT process, which makes the cells migratory and invasive and, thus, promote cancer metastasis (16). Similarly, the Wnt/β-Catenin signaling pathway drives tumor initiation and progression and is required for biological processes, such as cell proliferation, migration, invasion, and angiogenesis (17). Furthermore, β-Catenin translocation from the cytoplasm to the nucleus activates the target genes MMP7 and C-MYC, thereby playing an active role in tumor metastasis (18). Moreover, the Wnt/β-Catenin signaling pathway has been shown to promote tumor metastasis by regulating the EMT process in thyroid and pancreatic cancers (19, 20).

Genes have been reported to promote osteosarcoma progression through the TGF-β signaling pathway (21) or Wnt/β-Catenin signaling pathway (22) and are associated with the prognosis of osteosarcoma patients. This study aimed to identify new prognostic key factors associated with tumor metastasis for osteosarcoma. The samples were divided into metastatic and non-metastatic groups, and genes associated with osteosarcoma prognosis were identified through differential analysis, PPI, and LASSO-COX analysis. Subsequently, *in vitro* experiments were used to validate the expression and explore the biological functions of the key and further investigate the mechanisms of key genes on osteosarcoma metastasis.

## Materials and Methods

### Data Source and Processing

Clinical samples and RNA-seq data of 89 cases of osteosarcoma were obtained from the Target database (https://ocg.cancer.gov/programs/target). Human osteosarcoma MG-63, Saos-2, and U-2OS cell lines—which were purchased from the American Typical Culture Collection (ATCC, USA)—were used for *in vitro* experiments. Data were statistically analyzed using SPSS 17.0, and results are expressed as x ± s. Component data were compared using a t-test, and P < 0.05 was considered as a statistically significant difference in data.

### Analysis of Variance With Protein-Protein Interaction Network (PPI)

The samples were divided into transferred and non-transferred groups and analysis of variance (ANOVA) was performed using the R package “limma,” with |log2 FC| > 0.3785 and P < 0.05 as the screening condition. The STRING database (https://string-db.org/) was used to obtain the reciprocal network relationships among differentially expressed genes (DEGs) and then imported into Cytoscape software to eliminate isolated nodes and use the MCC algorithm to derive the top-10 ranked genes.

### Construction of a Prognostic Model

The LASSO-COX prognostic model was constructed by the R package “glmnet” to obtain a risk score formula containing multiple genes, and the samples were divided into two groups—high and low risk—in accordance with the calculation results. In addition, the R package “survivor” was used to perform survival analysis, aLog-rank was used to test the survival difference between high and low-risk groups, and the prediction accuracy of the model was analyzed using time ROC. The genes included in the model were subsequently analyzed using multifactorial COX regression, and genes with P < 0.05 were used as the key genes. The R package “ggplot2” was used to obtain the expression distribution of the key genes in the transferred and non-transferred groups, and the differences between the two groups were analyzed using the Wilcoxon rank-sum test.

### Cell Culture and Transfection

The human osteosarcoma MG-63, Saos-2, and U-2OS cell lines were cultured in a DMEM medium containing 10% FBS, and the cells were cultured and passaged at 37°C in an atmosphere of 5% CO2. In addition, MG-63 cells in the logarithmic growth phase were taken and divided into control, OE-COL5A2, and si-COL5A2 groups for transfection; the cells were collected for subsequent experiments 48h after transfection.

### Quantitative Real-Time PCR (qRT-PCR) for the Detection of Key Gene Expression

The transfected MG-63 cells were treated with Trizol reagent and RNA was extracted. cDNA was synthesized using a reverse transcription kit according to the manufacturer’s instructions. qRT-PCR was performed to amplify the reaction products, using GAPDH as the internal reference and the primer sequences were GAPDH, forward primer: CTGGGCTACACTGAGCACC, and the results were expressed as 2-∆∆Ct. The reaction products were synthesized by qRT-PCR with GAPDH as the internal reference and the primer sequences were GAPDH, forward primer: CTGGGCTACACTGAGCACC, reverse primer: AAGTGGTCGTTGAGGGCAATG; and COL5A2, forward primer: GACTGTGCCGACCCTGTAAC, reverse primer: CCTGGACGACCACGTATGC.

### Immunofluorescence (IF) Analysis

The transfected MG-63, Saos-2, and U-2OS cells were washed with PBS, fixed with 4% paraformaldehyde for 20 min, and then the cells were incubated in 0.3% Triton X-100 for 20 min. The closure was performed at room temperature, followed by immunofluorescence staining with primary antibody COL5A2 (ab7046,abcam). Cells were washed with PBS and incubated again with fluorescently labeled secondary antibodies and, finally, images were obtained using fluorescence microscopy.

### Western Blotting (WB)

MG-63 cells at the logarithmic growth stage were taken, cells were lysed with lysis buffer, and total proteins were extracted. The total protein concentration was determined using the BCA kit. Thereafter, protein samples were subjected to SDS-PAGE and transferred to PVDF membranes using anti-COL5A2 (ab7046, abcam), β-catenin (ab32572, abcam), Vimentin (ab92547, abcam), E-cadherin (ab233611, abcam), and N-cadherin (ab254512, abcam); these samples were incubated overnight at 4°C and then the membranes were immunoblotted with horseradish peroxidase-coupled secondary antibodies. β-actin (ab8226,abcam) was used as a control and the protein signal was detected using a gel imaging system.

### CCK-8 Detection oCell Proliferation

The transfected MG-63 cells were digested with trypsin and inoculated in 96-well plates, added with DMEM medium, and incubated at 37°C in an incubator with 5% CO2. According to the manufacturer’s instructions, CCK-8 solution was added after 0h, 6h, 12h, 24h, and 48h of incubation, respectively, and the incubation was continued for 3h. The absorbance value (OD) was measured at a wavelength of 450 nm; three experiments were performed in parallel, and the average value was taken for the calculation.

### Scratch Assay to Detect Cell Migration

The transfected human osteosarcoma MG-63 cells were inoculated in a six-well plate. When the cell density reached 90%, a 20 μL pipette tip was used to gently scrape the bottom of the well. The cells were washed using PBS solution, the scratched-down cells were removed, serum-free medium was added and incubated at 37°C in a 5% CO2 incubator, and the degree of healing of the scratches was observed at 0h and 24h, respectively. The cell migration rate (%) = (initial scratch width value - corresponding time point scratch width value)/initial scratch width value × 100%.

### Transwell Assay for Cell Invasion

Transfected human osteosarcoma MG-63 cells were taken and inoculated into the upper chamber of transwell coated with Matrigel matrix gel, and serum-free medium was added to maintain cell survival. Medium containing 10% FBS was added to the lower chamber of the transwell as a chemical elicitor, and the cells remaining on the membrane surface were removed after incubation for 48h. The membranes were fixed with 100% ethanol for 5 min and then stained with 5% crystalline violet for 5 min. Photographs were taken using a light microscope, and the cell numbers were counted and averaged.

## Results

### Preliminary Gene Screening

**Figure 1:**
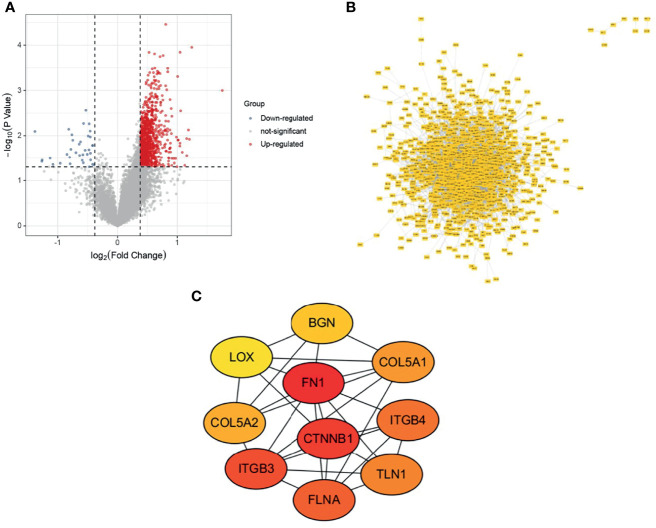
Variance analysis and PPI construction. **(A)** Volcano map; **(B)** PPI network map; **(C)** Top 10 genes.

The osteosarcoma samples in the Target database were divided into metastatic and non-metastatic groups for differential gene expression analysis, and 1100 up-regulated genes and 36 down-regulated genes were obtained (**Figure 1A**). PPI networks were constructed using DEGs, which were used to observe the protein-protein interactions (**Figure 1B**). The top 10 key genes (COL5A2, BGN, ITGB4, COL5A1, ITGB3, TLN1, FLNA, CTNNB1, FN1, LOX) were subsequently obtained by the MCC algorithm (**Figure 1C**).

### COL5A2: A Key Gene Related to the Prognosis of Osteosarcoma

**Figure 2:**
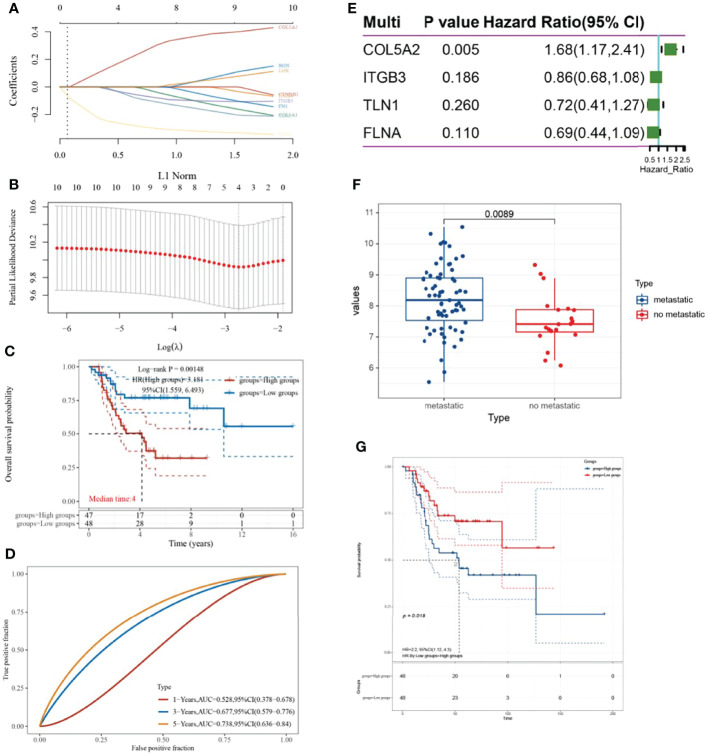
Establishment of the LASSO-COX prognostic model and screening of key genes. **(A, B)** λ parameters for selected features and partial likelihood deviation plotted against log(λ) using the LASSO-COX model; **(C)** KM survival curve based on risk grouping; **(D)** time ROC curve; **(E)** forest plot; **(F)** box line plot with COL5A2 expression distribution based on the presence or absence of metastatic grouping; **(G)** relationship between high and low COL5A2 expression and prognosis of osteosarcoma.

A LASSO-COX prognostic risk model was constructed based on the top-10 ranked key genes, and a risk scoring system was obtained when the minimum λ was 0.0645 (**Figure 2A, B**). The corresponding risk score values were calculated based on risk score = (0.1982)*COL5A2 + (-0.043) × ITGB3 + (-0.0562) × TLN1 + (-0.2716) × FLNA, and the samples were divided into high-risk and low-risk groups. KM survival curves reveal that the high-risk group was associated with a poor prognosis of osteosarcoma (P = 0.00148, HR = 3.181) (**Figure 2C**). Furthermore, the ROC curve revealed that the risk model had a good predictive ability (**Figure 2D**). Multi-factorial COX regression analysis of four genes in the risk model related to the prognosis of osteosarcoma led to the identification of COL5A2 as the key gene associated with osteosarcoma prognosis (**Figure 2E**). COL5A2 was significantly expressed in the osteosarcoma metastasis group (P = 0.0089) and its high expression led to a worse prognosis (P = 0.018, HR = 2.2) (**Figures 2F** **, G**).

### High Expression of COL5A2 in Osteosarcoma Cells

**Figure 3:**
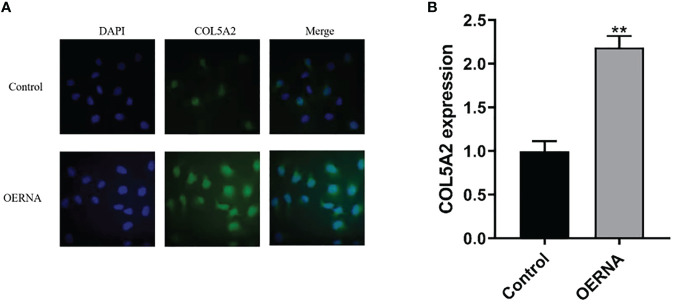
COL5A2 expression validation. **(A)** Immunofluorescence to verify the differential expression of COL5A2 in different types of osteosarcoma cell lines up to; **(B)** qRT-PCR to verify the high and low expression levels of COL5A2 in osteosarcoma **P<0.01.

To determine the relationship between COL5A2 and osteosarcoma progression, this study first verified the expression of COL5A2 using qRT-PCR and immunofluorescence techniques. As shown in the figure, the expression of col5a2 in human osteosarcoma MG-63 cells was analyzed by immunofluorescence technique (**Figure 3A**). Subsequently, human osteosarcoma MG-63 cells were divided into control, COL5A2-enhanced, and COL5A2-knockdown groups and the different expression levels of COL5A2 in MG-63 cells were verified using qRT-PCR with GAPDH as the control gene (**Figure 3B**). In addition, the results of this experiment demonstrated that the cells were successfully transfected.

### The Pro-Tumor Effect of COL5A2

**Figure 4:**
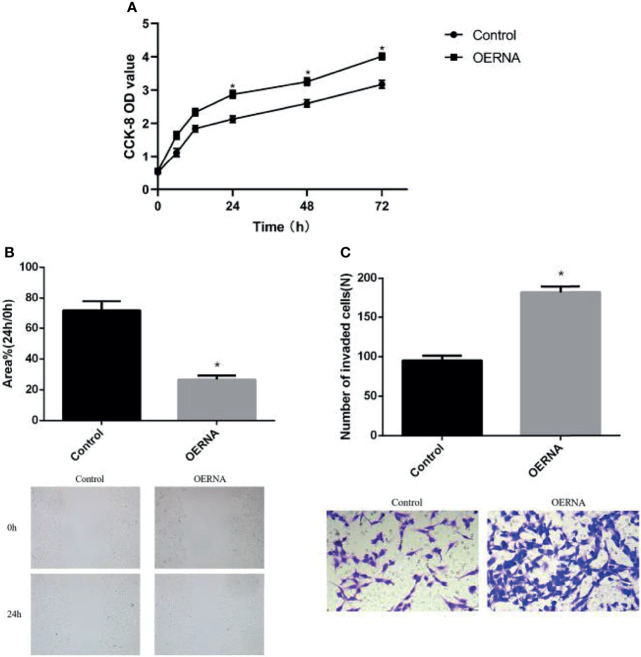
High expression of COL5A2 promotes the progression of osteosarcoma. **(A)** Enhanced promotion of osteosarcoma cell proliferation by COL5A2 (CCK-8 assay) (P < 0.05); **(B)** scratch assay to observe the migration ability of osteosarcoma cells after the upregulation of COL5A2 expression *P<0.05; **(C)** transwell assay was used to assess the invasiveness of osteosarcoma cells after the upregulation of COL5A2 expression *P<0.05.

In order to investigate the biological role of COL5A2 in osteosarcoma, OERNA with COL5A2-enhancing effect was transfected, and its expression was stably upregulated in MG-63 cells, which is a finding verified in **Figure 3B**. The proliferation rate was significantly faster in the experimental group compared to the control group and revealed a significant difference from 6h onward (**Figure 4A**). Similarly, the migration ability of osteosarcoma cells was enhanced after transfection with OERNA of COL5A2 (**Figure 4B**). In addition, the Transwell assay revealed that the upregulation of COL5A2 promoted the invasive effect of osteosarcoma cells (**Figure 4C**). All these experiments demonstrated that the upregulation of COL5A2 expression contributes to the promotion of osteosarcoma progression.

### COL5A2 Can Affect Osteosarcoma Progression Through the Wnt/β-Catenin and TGF-β Signaling Pathways

**Figure 5:**
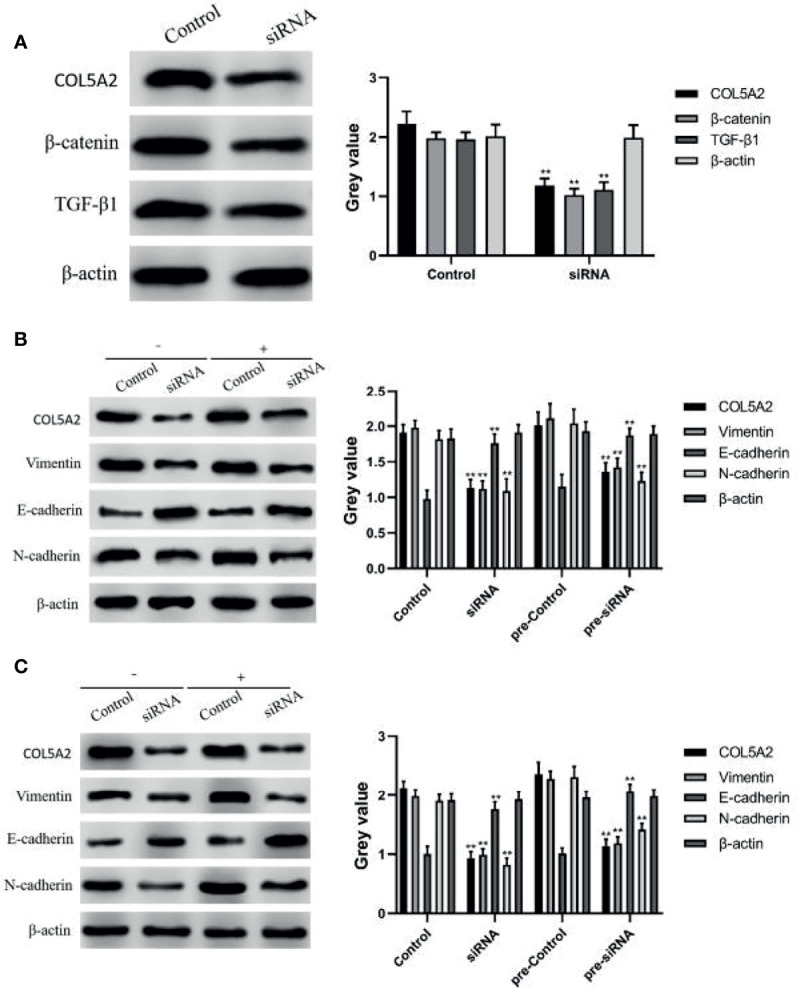
WB to verify the relationship between COL5A2 and signaling pathways. **(A)** Expression of β-catenin, TGFF-β1 was detected by WB after reducing the expression of COL5A2 **P<0.01. **(B)** protein expression levels of COL5A2, E-cadherin, N-cadherin, and Vimentin were assessed by WB after treatment with or without TGF-β1 **P<0.01. **(C)** protein expression levels of COL5A2, E-cadherin, N-cadherin, and Vimentin were assessed by WB after treatment with or without Wnt/β-catenin treatment and the protein expression levels of COL5A2, E-cadherin, N-cadherin, and Vimentin **P<0.01.

TGF-β signaling and Wnt/β-catenin signaling can promote the EMT process by increasing the expression of N-cadherin and Vimentin or decreasing the expression of E-cadherin, thereby promoting tumor invasion and metastasis (15, 23). In this study, we first examined the expression of Wnt/β-catenin and TGF-β pathway proteins by WB; the results revealed that the knockdown of COL5A2 decreased the expressions of β-catenin and TGF-β1 (**Figures 5A**). Subsequently, the experimental subjects were divided into two groups treated with or without TGF-β1, each comprising a control group and a si-COL5A2 group. WB analysis revealed that N-cadherin and Vimentin expression was downregulated by the knockdown of COL5A2, while E-cadherin expression was upregulated with the knockdown of COL5A2 (**Figures 5B**). Using the same method to detect the effect of COL5A2 knockdown on the Wnt/β-catenin signaling pathway again, the same conclusion can be drawn (**Figures 5C**). These results suggest that the downregulation of COL5A2 can promote osteosarcoma metastasis by affecting either the Wnt/β-catenin signaling pathway or the TGF-β signaling pathway.

## Discussion

Osteosarcoma is an important threat to the health of children and adolescents. Currently, surgical resection is often used to control local tumors at the primary tumor site, while neoadjuvant multidrug chemotherapy is mostly used for unresectable cases, like distant metastases (24, 25). However, the high propensity to metastasize leads to a high mortality rate and often a poor prognosis for patients with osteosarcoma (26). Studies have revealed that several proteins or RNAs are tumor-promoting factors in osteosarcoma and they play a regulatory or inducing role in tumor progression (27, 28). This study aimed to identify new prognostic factors associated with osteosarcoma metastasis and to briefly describe the mechanisms by which they affect cancer cell progression.

Osteosarcoma samples were grouped based on whether or not they metastasized and screened by bioinformatics analysis to obtain COL5A2. Type V α2 collagen (COL5A2) is mainly found in the basement membrane of the skin and provides a template for the composition of type V collagen (29). It was revealed that the upregulation of the collagen family gene (COL) can be used as a diagnostic marker for osteosarcoma (30). In contrast, COL5A2, a member of the collagen family, is involved in immune system regulation, angiogenesis, and tumor metastasis, and plays a role in promoting tumor progression (31, 32). It has been demonstrated that high COL5A2 expression promotes the proliferation and invasion of prostate cancer cells (33), is associated with poor prognosis in patients with gastric cancer (34), and promotes the proliferation of colon cancer cells by activating the Wnt/β-catenin signaling pathway (35). In this study, the analysis of bioinformatics revealed that COL5A2 indicated a high expression status in metastatic osteosarcoma and led to a poor prognosis. To explore the role and functions played by COL5A2 in osteosarcoma progression, a series of *in vitro* experiments were performed in this study. qRT-PCR and immunofluorescence assays confirmed the high expression of COL5A2 in human osteosarcoma cells. Subsequent CCK-8 assays, scratch assays, and transwell assays confirmed that enhanced COL5A2 expression contributes to the proliferation, invasion, and migration of osteosarcoma cells. These results suggest that high COL5A2 expression may be associated with the high metastatic properties of osteosarcoma.

In the current study, EMT is considered to be a key factor involved in tumor cell invasion and metastasis, and it has been reported that metastasis can be promoted by regulating the EMT process in colorectal cancer (23), breast cancer (36), and head and neck squamous cell carcinoma (37). Moreover, in osteosarcoma, the EMT process is also associated with tumor cell metastasis (38). The tGF-β signaling and Wnt/β-catenin signaling pathways are two important pathways that activate the EMT process and are both involved in tumor invasion and metastasis (39, 40). tGF-β is an important factor that regulates the immune response *in vivo*, and tumor cells can manipulate the regulatory process of TGF-β. TGF-β can lead to cell cycle arrest and apoptosis in the early stages of cancer, but cancer cells usually transform TGF-β signaling into a tumor-promoting function by mutating key components of the TGF-β signaling pathway (41). Dysregulation of the TGF-β signaling pathway is often a signal for tumorigenesis. In patients with osteosarcoma, the upregulation of TGF-β expression can lead to increased chemoresistance and subsequently poor prognosis (42). Similarly, there are numerous studies that confirm that the Wnt/β-catenin signaling pathway is one of the major oncogenic pathways involved in the progression of human osteosarcoma (43). β-catenin is a key mediator of Wnt signaling and contributes to stable cell-to-cell contacts (44). An aberrant Wnt/β-catenin signaling pathway promotes cancer stem cell renewal, cell proliferation, and differentiation as well as plays an important role in tumorigenesis (45). The invasive and metastatic process of several cancers can be promoted by activating the Wnt/β-catenin signaling pathway (46, 47). Further, different genes have been shown to inhibit the growth of osteosarcoma by suppressing the TGF-β signaling pathway or the Wnt/β-catenin signaling pathway (48, 49). In this study, we further determined that the knockdown of COL5A2 expression inhibited the TGF-β signaling and Wnt/β-catenin signaling pathways, thereby suppressing the invasion and migration of osteosarcoma.

In the present study, COL5A2 was the only gene screened for prognostic relevance in patients with osteosarcoma based on whether they were grouped metastatically. COL5A2 is highly expressed in patients with metastatic osteosarcoma and indicates a poor prognostic survival. In the study by Chen et al., COL5A2 was investigated as a gene regulatory mediator and the authors simply demonstrated that the knockdown of COL5A2 inhibited metastasis and proliferation of osteosarcoma (50). This is consistent with our findings that reveal that enhanced COL5A2 expression accelerated the proliferation, invasion, and migration rate of osteosarcoma cells. Furthermore, WB experiments confirmed that COL5A2 could achieve tumor suppression by inhibiting the TGF-β signaling or Wnt/β-catenin signaling pathways.

## Data Availability Statement

The original contributions presented in the study are included in the article/supplementary material. Further inquiries can be directed to the corresponding author.

## Author Contributions

Y-LH, DL, KH, WA, and LW: Conceptualization, Methodology, Software. Y-LH, DL and LW: Data curation, Writing- Original draft preparation. WW: Visualization, Investigation. JT, WZ, WW and WA: Software, Validation. Y-LH and LW: Writing- Reviewing and Editing.

## Conflict of Interest

The authors declare that the research was conducted in the absence of any commercial or financial relationships that could be construed as a potential conflict of interest.

## Publisher’s Note

All claims expressed in this article are solely those of the authors and do not necessarily represent those of their affiliated organizations, or those of the publisher, the editors and the reviewers. Any product that may be evaluated in this article, or claim that may be made by its manufacturer, is not guaranteed or endorsed by the publisher.
